# Supplementary material for: Social Frailty and Social Isolation in Chinese Community-Dwelling Older Adults: A Network Analysis
Source: Nurs Rep. 2025 Aug 27;15(9):315. doi: 10.3390/nursrep15090315 (PMC12472408; doi:10.3390/nursrep15090315)

Supplementary Figure S3. Correlation stability coefficient of symptom network in participants.

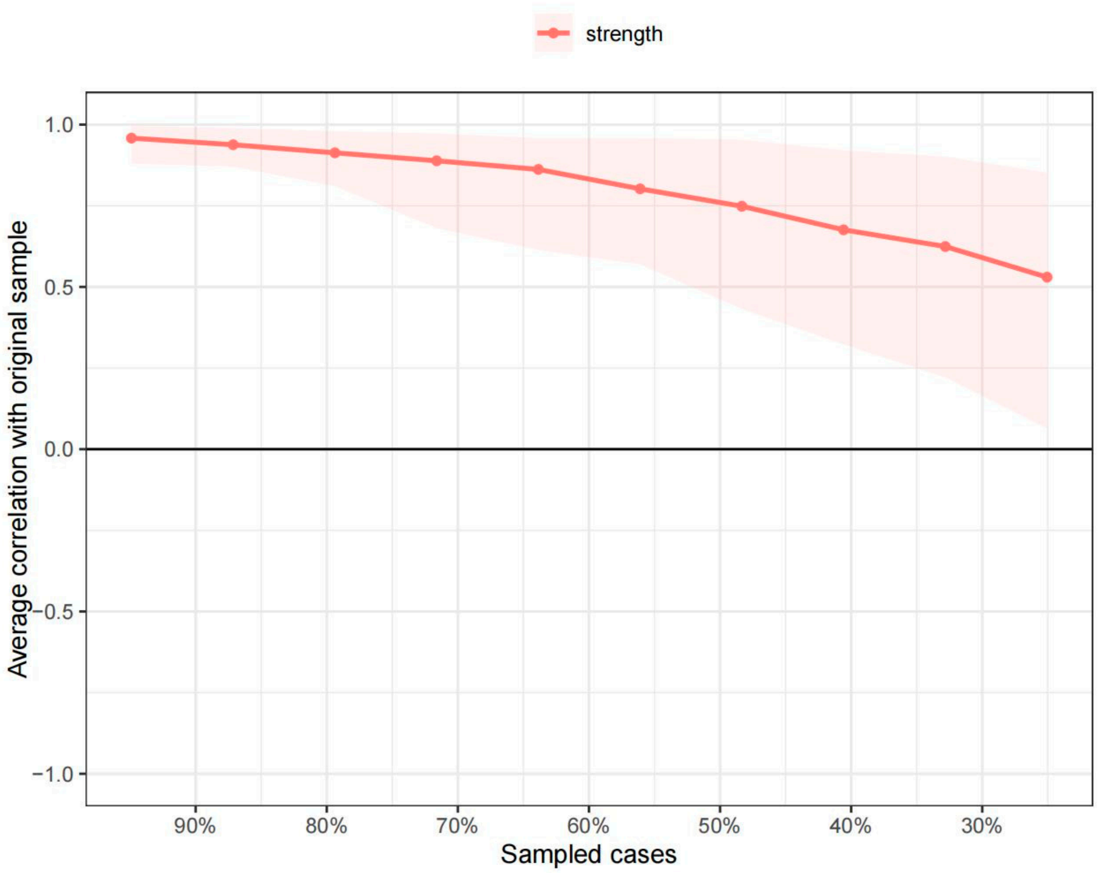

Supplement: Supplementary file 1 [file nursrep-15-00315-s001.zip › Supplementary figure S3.pdf]
