# Supplementary material for: Social Frailty and Social Isolation in Chinese Community-Dwelling Older Adults: A Network Analysis
Source: Nurs Rep. 2025 Aug 27;15(9):315. doi: 10.3390/nursrep15090315 (PMC12472408; doi:10.3390/nursrep15090315)
Supplement: Supplementary file 1 [file nursrep-15-00315-s001.zip › Supplementary table S1.pdf]

Supplementary Table S1. The correlation matrix of the social frailty and social isolation network of participants.

	**SF1**	**SF2**	**SF3**	**SF4**	**SF5**	**SI1**	**SI2**	**SI3**	**SI4**	**SI5**	**SI6**
**SF1**	0.0000000	1.8495324	0.6809273	0.0000000	0.4406360	0.0000000	0.0000000	0.0000000	-0.2290375	0.0000000	0.0000000
**SF2**	1.8495324	0.0000000	0.7757223	0.0000000	0.5365439	0.0000000	0.0000000	0.0000000	0.0000000	0.0000000	0.0000000
**SF3**	0.6809273	0.7757223	0.0000000	0.0000000	0.2686672	0.0000000	0.0000000	0.0000000	0.0000000	0.0000000	0.0000000
**SF4**	0.0000000	0.0000000	0.0000000	0.0000000	1.0594123	0.0000000	0.0000000	0.0000000	-0.2727786	0.0000000	0.0000000
**SF5**	0.4406360	0.5365439	0.2686672	1.0594123	0.0000000	0.0000000	-1.1081742	0.0000000	0.0000000	0.0000000	0.0000000
**SI1**	0.0000000	0.0000000	0.0000000	0.0000000	0.0000000	0.0000000	0.8797180	1.1828495	0.4821000	0.1578255	0.0000000
**SI2**	0.0000000	0.0000000	0.0000000	0.0000000	-1.1081742	0.8797180	0.0000000	1.0964160	0.1212646	0.6521251	1.1980579
**SI3**	0.0000000	0.0000000	0.0000000	0.0000000	0.0000000	1.1828495	1.0964160	0.0000000	0.8423418	0.0000000	0.0000000
**SI4**	-0.2290375	0.0000000	0.0000000	-0.2727786	0.0000000	0.4821000	0.1212646	0.8423418	0.0000000	1.7320931	0.3752635
**SI5**	0.0000000	0.0000000	0.0000000	0.0000000	0.0000000	0.1578255	0.6521251	0.0000000	1.7320931	0.0000000	0.5027682
**S16**	0.0000000	0.0000000	0.0000000	0.0000000	0.0000000	0.0000000	1.1980579	0.0000000	0.3752635	0.5027682	0.0000000
